# Signature program: a platform of basket trials

**DOI:** 10.18632/oncotarget.25109

**Published:** 2018-04-20

**Authors:** Eric D. Slosberg, Barinder P. Kang, Julio Peguero, Matthew Taylor, Todd M. Bauer, Donald A. Berry, Fadi Braiteh, Alexander Spira, Funda Meric-Bernstam, Steven Stein, Sarina A. Piha-Paul, August Salvado

**Affiliations:** ^1^ Novartis Pharmaceuticals Corporation, East Hanover, NJ, USA; ^2^ Oncology Consultants PA, Houston, TX, USA; ^3^ Oregon Health and Science University, Portland, OR, USA; ^4^ Sarah Cannon Research Institute/Tennessee Oncology, PLLC, Nashville, TN, USA; ^5^ The University of Texas MD Anderson Cancer Center, Houston, TX, USA; ^6^ Berry Consultants, Austin, TX, USA; ^7^ US Oncology Research and Comprehensive Cancer Centers of Nevada, Las Vegas, NV, USA; ^8^ Virginia Cancer Specialists, Fairfax, VA, USA; ^9^ Incyte, Wilmington, DE, USA; ^10^ Current affiliation: Daiichi Sankyo, Inc, Basking Ridge, NJ, USA

**Keywords:** signature, clinical trial design, basket trial, mutations, tissue agnostic

## Abstract

Investigating targeted therapies can be challenging due to diverse tumor mutations and slow patient accrual for clinical studies. The Signature Program is a series of 8 phase 2, agent-specific basket protocols using a rapid study start-up approach involving no predetermined study sites. Each protocol evaluated 1 agent (buparlisib, dovitinib, binimetinib, encorafenib, sonidegib, BGJ398, ceritinib, or ribociclib) in patients with solid or hematologic malignancies and an actionable mutation. The primary endpoint of each study was the clinical benefit rate (ie, complete or partial response, or stable disease) at 16 weeks. A total of 192 individual sites were opened in the United States, with a median start-up time of 3.6 weeks. The most common tumor types among the 595 treated patients were colorectal (9.2%), non-small cell lung adenocarcinoma (9.1%), and ovarian (8.4%). Frequent genetic alterations were in *PIK3CA*, *RAS*, *p16*, and *PTEN*. Overall, 30 partial or complete responses were observed with 6 compounds in 16 tumor types. The Signature Program presents a unique and successful approach for rapid signal finding across multiple tumors and allowed various agents to be evaluated in patients with rare alterations. Incorporating these program features in conventional studies could lead to improved trial efficiencies and patient outcomes.

## INTRODUCTION

Genetic profiling has become readily accessible to oncologists in most practice settings in the United States. This technology can identify potentially actionable genetic alterations in a tumor, allowing physicians to match individual patients with a targeted therapy [1, 2]. Although agents targeting a wide range of molecules and pathways have been discovered, investigating these therapies can be challenging due to the diversity of molecular drivers, even within tumors of the same histology, and the rarity of these patients at any one hospital or clinic. This results in operational inefficiencies, slow patient accrual, and long timelines for clinical trials [2–4]. One approach to match patients with genetic alterations to targeted therapies is the basket trial. Basket trials use a hypothesis-generating approach in which enrollment and treatment assignment are molecularly driven [3], and a single targeted agent is tested simultaneously in tumors of different histologies that have genetic alterations in the targeted pathway [5].

**Figure 1:**
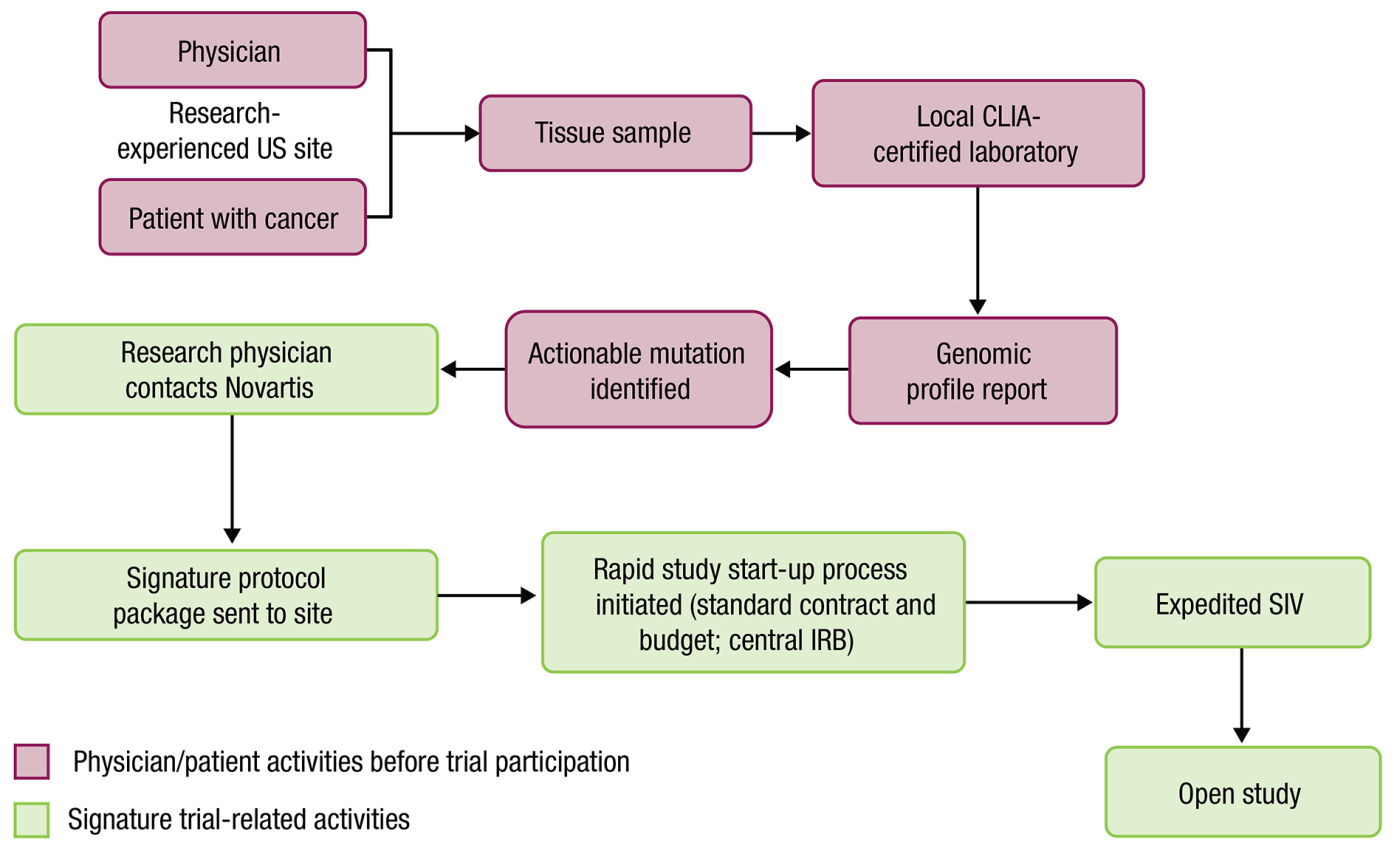
Signature Program protocol start-up process Each protocol excluded patients with certain tumor types, including those for which the agent being studied has shown no benefit and those for which key studies are planned or ongoing. CLIA, Clinical Laboratory Improvement Amendments; IRB, institutional review board; SIV, site initiation visit.

Novartis Pharmaceuticals Corporation built on this approach and launched the Signature Program, a series of 8 agent-specific, open-label, phase 2 basket trials in which research-qualified academic or community physicians in the United States could participate (Table 1). Each trial investigated a single agent, and enrollment was based on tissue-agnostic, molecularly driven criteria. The statistical design was similar for each protocol and used a patient-sparing approach and a Bayesian adaptive design [6]. Importantly, participating sites were not predetermined but were rapidly opened after a potentially eligible patient was identified (Figure 1).

**Table 1:** Agents included in the Signature Program

Agent	Target	Mutations Required	Tumor Types Excluded	ClinicalTrials.gov ID
Buparlisib (BKM120) [28]	Pan-Pl3K	*PIK3CA* mutation/amplification, *PTEN* mutation/loss, or *PIK3R1* mutation	Endometrial, glioblastoma, NSCLC, prostate, breast	NCT01833169
Dovitinib (TKI258) [29]	Various RTKs	*FGFR1-3*, *FLT3*, or *c-KIT* mutation/amplification or *PDGFRα*/*β*, *VEGFR1-2*, *RET*, *TrkA* (*NTRK1*), or *CSF-1R* mutation	Multiple myeloma, urothelial, AML (FLT3+), hepatocellular, endometrial, renal cell, breast (metastatic), squamous NSCLC	NCT01831726
Binimetinib (MEK162)^a^ [30]	MEK (RAS pathway)	*RAS*, *RAF*, *MEK1*/*MEK2*, or *NF1*	Pancreatic, biliary, colorectal, ovarian (low-grade serous), melanoma	NCT01885195
Encorafenib (LGX818)^a^ [31]	BRAF	*BRAF* V600E	Melanoma, colorectal, primary CNS	NCT01981187
Sonidegib (LDE225)^b^ [32]	SMO (hedgehog pathway)	*PTCH1* or *SMO*	Basal cell, pancreatic, medulloblastoma/primary CNS, CML, ALL, AML	NCT02002689
BGJ398 [33]	FGFR	*FGFR* mutation/amplification/fusion, *FGFR1-4* translocation, or ligand amplification	Urothelial, cholangiocarcinoma, glioblastoma multiforme	NCT02160041
Ceritinib (LDK378)^c^ [34]	ALK/ROS1	*ALK*/*ROS1* mutation/amplification/translocation/rearrangement	ALK+ NSCLC	NCT02186821
Ribociclib (LEE011)^d^ [35]	CDK4/6	*CDK4/6* mutation/amplification*, cyclin D1/D3* amplification, or *p16* mutation/loss	ER+ breast, mantle cell lymphoma, teratoma, liposarcoma, castration-resistant prostate, melanoma	NCT02187783

Abbreviations: ALK, anaplastic lymphoma kinase; ALL, acute lymphocytic leukemia; AML, acute myeloid leukemia; CDK, cyclin-dependent kinase; CML, chronic myeloid leukemia; CNS, central nervous system; CSF-1R, colony-stimulating factor 1 receptor; ER, estrogen receptor; FGFR, fibroblast growth factor receptor; FLT3, fms-related tyrosine kinase 3; MEK, mitogen-activated protein kinase/extracellular signal–regulated kinase kinase; NF1, neurofibromatosis type 1; NSCLC, non-small cell lung cancer; NTRK1, neurotrophic tyrosine kinase receptor type 1; PDGFR, platelet-derived growth factor receptor; PI3K, phosphatidylinositol 3-kinase; PIK3CA, phosphatidylinositol 4,5-bisphosphate 3-kinase catalytic subunit α; PIK3R1, phosphatidylinositol 3-kinase regulatory subunit polypeptide 1; PTCH1, patched 1; PTEN, phosphatase and tensin homolog; RTK, receptor tyrosine kinase; SMO, smoothened; TrkA, tropomyosin receptor kinase A; VEGFR, vascular endothelial growth factor receptor.

^a^Binimetinib (MEK162) and encorafenib (LGX818) are owned by Array BioPharma.

^b^Sonidegib (LDE225; Odomzo), owned by Sun Pharmaceuticals, is a hedgehog pathway inhibitor indicated for the treatment of adult patients with locally advanced basal cell carcinoma that has recurred following surgery or radiation therapy or those who are not candidates for surgery or radiation therapy [36].

^c^Ceritinib (LDK378) is approved by the US Food and Drug Administration for the second-line treatment of ALK+ NSCLC [37].

^d^Ribociclib (LEE011; Kisqali) was discovered by the Novartis Institutes for Biomedical Research in collaboration with Astex Pharmaceuticals. Kisqali is a kinase inhibitor indicated in combination with an aromatase inhibitor as initial endocrine-based therapy for the treatment of postmenopausal women with hormone receptor–positive, human epidermal growth factor receptor 2–negative advanced or metastatic breast cancer [38].

The Signature Program had 4 key objectives: (1) rapidly match each patient with a treatment that targets the tumor's molecular abnormality, (2) discover the clinical potential of pipeline compounds by matching the drug's mechanism of action to molecular targets across a range of malignancies, (3) develop a better understanding of the underlying disease biology of the individual patient, and (4) develop a platform that can accelerate downstream clinical development by rapidly focusing new targeted agents on indications with the clearest signals for further tissue-specific trials. Herein, we present an overview of this novel clinical trial approach and report the overall findings from the program. Analyses of each individual protocol will be reported in subsequent publications.

## RESULTS

As of the data cutoff (December 18, 2017 [October 1, 2015, for binimetinib]), 1674 prescreening checklists were received, 1568 patients had actionable mutations, and 988 patients provided informed consent. Overall, 595 patients received treatment. The main reasons for patient drop-off were unacceptable laboratory value(s) (116/414), unacceptable test procedure result(s) (57/414), and other (99/414), which included screening failure, initiation of an alternative therapy, expired consent, ineligible mutational status, and death. Patients were enrolled at 192 individual sites (396 protocol openings) including research networks, community sites, and academic institutions (Table 2). Each arm was considered a separate protocol, resulting in site overlap and the possibility for individual sites to be counted twice. The median start-up time was 3.6 weeks (range, 0.3-35.9 weeks) across all sites (mean, 5.9 weeks) and was shortest for research networks (2.6 weeks) and longest for academic institutions (8.1 weeks).

**Table 2:** Signature Program enrollment

Site type	Sites, n (%)^a^	Time to trial start, weeks			Patients treated, n
		Mean	Median	Range	
Research network	118 (30)	3.6	2.6	0.3-34.8	166
Community site	197 (50)	5.4	3.6	1.0-35.9	196
Academic	81 (20)	10.7	8.1	1.4-33.7	233
Total	396	5.94	3.6	0.3-35.9	595

^a^Percentage among total protocol openings (N = 396) at 192 individual sites (a single site may have opened > 1 protocol).

Across all protocols, the median patient age was 61 years, and the median number of prior lines of drug therapy was 3 (range, 0-19) (Table 3). The most commonly enrolled tumor types were colorectal (n = 55 [9.2%]), non-small cell lung cancer (NSCLC) adenocarcinoma (n = 54 [9.1%]), ovarian (n = 50 [8.4%]), sarcoma (n = 45 [7.6%]), head and neck squamous cell carcinoma (n = 32 [5.4%]), and uterine (n = 20 [3.4%]). Patients with a variety of rarer tumor types (eg, gallbladder/biliary [n = 17 (2.9%)], anal [n = 10 (1.7%)], mesothelioma [n = 6 (1.0%)], vaginal [n = 5 (0.8%)], germ cell [n = 3 (0.5%)], thymus [n = 4 (0.7%)], and penile [n = 2 (0.3%)]) also were enrolled. By the data cutoff, tumor-type cohorts were formed for all agents except sonidegib, as only 10 patients were accrued over a period of 11 months (Table 4). In an average of 9.1 months, 13 cohorts reached the futility analysis target accrual of ≥ 10 patients at 16 weeks. Timing was based on the date of the first dose received by the first and last patients in a cohort. Overall, 4 of these cohorts closed due to futility (Table 4).

**Table 3:** Demographics of enrolled patients

	Buparlisib (BKM120)	Dovitinib (TKI258)	Binimetinib (MEK162)	Encorafenib (LGX818)	Sonidegib (LDE225)	BGJ398	Ceritinib (LDK378)	Ribociclib (LEE011)	Total
Patients treated, n	146	80	110	12	10	84	47	106	595
Age, median, years	60	60	62	58	67	61	58	63	61
Male, %	42	50	38	42	40	44	53	47	44
White, %	89	86	86	92	60	88	81	85	86
ECOG PS, %^a^									
0	37	48	35	58	20	25	40	34	36
1	63^b^	53	65	42	80	75	60	66	64^b^
Prior lines of therapy, median (range), n^c^	3 (1-13)	4 (0-14)	3 (0-16)	2 (0-7)	4 (1-11)	3 (0-14)	3 (0-14)	3 (0-19)	3 (0-19)

Abbreviations: ECOG, Eastern Cooperative Oncology Group; PS, performance status.

^a^May not equal 100% due to rounding.

^b^Includes 1 patient with ECOG PS of 2 at baseline.

^c^Only prior drug therapies are included.

**Table 4:** Tumor cohorts (n ≥ 4 patients)

Agent^a^	Buparlisib (BKM120)	Dovitinib (TKI258)	Binimetinib (MEK162)	Encorafenib (LGX818)	BGJ398	Ceritinib (LDK378)	Ribociclib (LEE011)
Cohorts	· Colorectal^b^	· GIST	· NSCLC (adeno)^b^	· Thyroid	· Breast	· Colorectal	· NSCLC (adeno)
	· Sarcoma^b^	· Colorectal	· Ovarian		· Colorectal	· NSCLC	· HNSCC
	· Ovarian^b^	· Ovarian	· Uterine		· HNSCC	(adeno)	· Sarcoma
	· Cervical	· Adenoid	· Appendix		· NSCLC	· Sarcoma	· Uterine
	· HNSCC	cystic	· Small intestine		(adeno)		· NSCLC
	· Anal	· HNSCC	· Sarcoma		· Ovarian		(squamous)
	· Gallbladder	· NSCLC	· Thyroid				· Breast (triple negative)
	· Bladder	(adeno)	· Unknown primary				
	· Gallbladder	· Thymus	· Breast				· Mesothelioma
	duct		· Bladder				· Pancreatic
	· GE junction		· GE junction				· Bladder
	· Liver		· Neuroendocrine				· GE junction
	· Skin						· Unknown primary
	nonmelanoma						· Head and neck
	· Small intestine						(nonsquamous)
	· Thyroid						
	· Unknown						
	primary						
	· Vaginal						
	· Neuroendocrine						

Abbreviations: CR, complete response; PR, partial response; SD, stable disease. adeno, adenocarcinoma; GE, gastroesophageal; GIST, gastrointestinal stromal tumor; HNSCC, head and neck squamous cell carcinoma; NSCLC, non-small cell lung cancer.

^a^No cohorts (defined as ≥ 4 patients treated) were formed for sonidegib (LDE225).

^b^Cohort was closed for futility.

**Figure 2:**
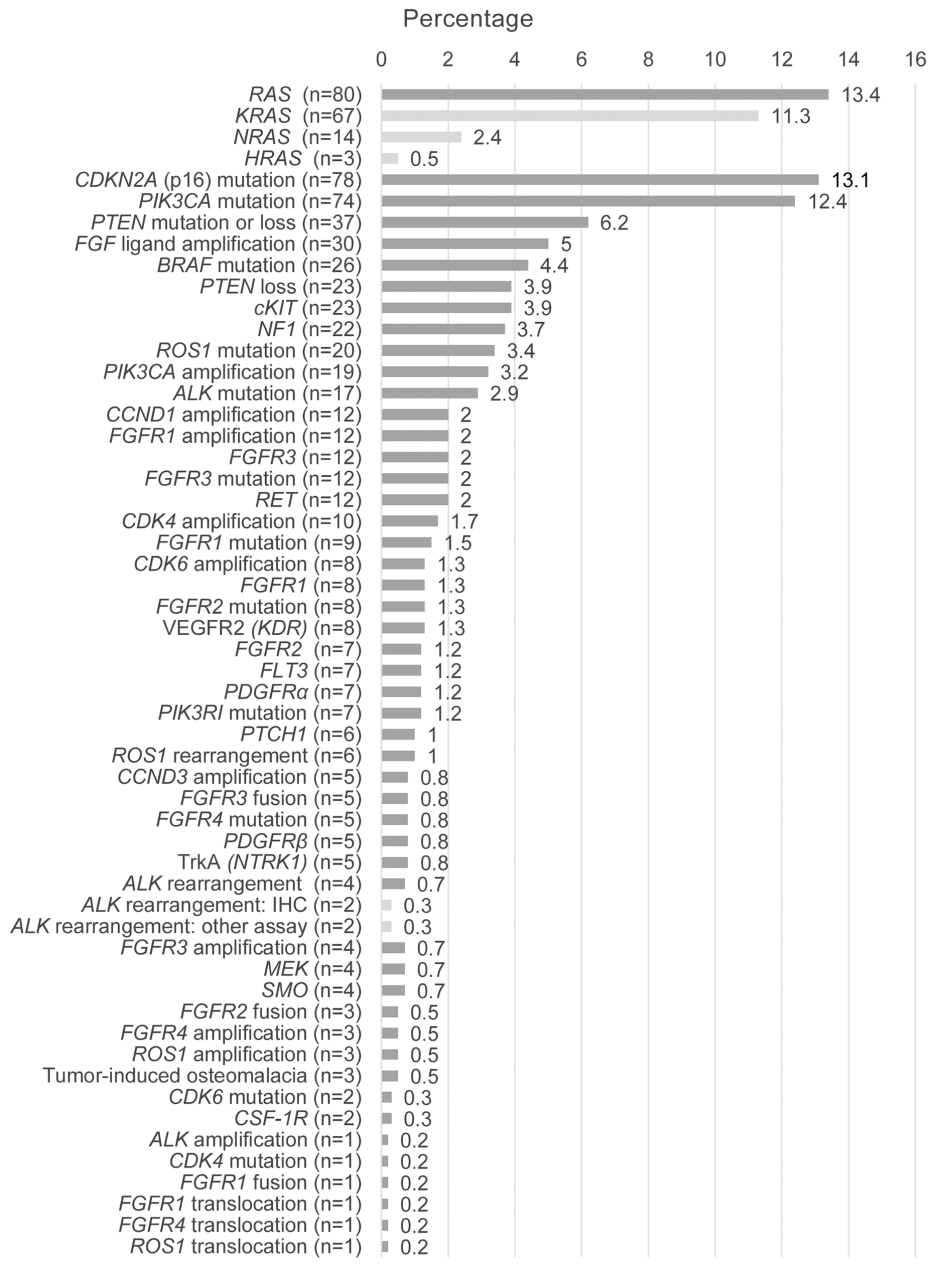
Summary of local alteration types (N = 595) ALK, anaplastic lymphoma kinase; CCND, cyclin D; CDK, cyclin-dependent kinase; CDKN2A, cyclin-dependent kinase inhibitor 2A; CSF-1R, colony-stimulating factor 1 receptor; FGF, fibroblast growth factor; FGFR, fibroblast growth factor receptor; FLT3, fms-related tyrosine kinase 3; HRAS, Harvey rat sarcoma viral oncogene homolog; IHC, immunohistochemistry; KDR, kinase insert domain receptor; KRAS, Kirsten rat sarcoma viral oncogene homolog; MEK, mitogen-activated protein kinase/extracellular signal–regulated kinase kinase; NF1, neurofibromatosis type 1; NRAS, neuroblastoma RAS viral oncogene homolog; NTRK1, neurotrophic tyrosine kinase receptor type 1; PDGFR, platelet-derived growth factor receptor; PIK3CA, phosphatidylinositol 4,5-bisphosphate 3-kinase catalytic subunit α; PIK3RI, phosphatidylinositol 3-kinase regulatory subunit polypeptide 1; PTCH1, patched 1; PTEN, phosphatase and tensin homolog; SMO, smoothened; TrkA, tropomyosin receptor kinase A; VEGFR, vascular endothelial growth factor. ^a^Patients may have been counted in > 1 category; ^b^PTEN loss determined by IHC (< 10% of tumor cells expressing PTEN at 1+ level); ^c^Referring to ALK positivity.

A wide array of genetic alterations were represented (Figure 2). The most frequent alterations (in ≥ 10% of patients) were phosphatidylinositol 4,5-bisphosphate 3-kinase catalytic subunit α (*PIK3CA*; n = 93 [15.6%]; gene mutation, n = 74; gene amplification, n = 19), *RAS* (n = 80 [13.4%]; Kirsten rat sarcoma viral oncogene homolog [*KRAS*], n = 67; neuroblastoma RAS viral oncogene homolog [*NRAS*], n = 14; Harvey rat sarcoma viral oncogene homolog [*HRAS*], n = 3; patients may have been counted in more than 1 *RAS* category), cyclin-dependent kinase inhibitor 2A encoding p16 (*CDKN2A*; n = 78 [13.1%]), and phosphatase and tensin homolog (*PTEN*; n = 60 [10.1%]; mutation/loss, n = 37; loss by immunohistochemistry, n = 23). The primary endpoint—clinical benefit rate (ie, partial response, complete response, or stable disease) at 16 weeks—was 17% (101/593; Table 5). Overall, 30 partial or complete responses (20 confirmed) were observed with 6 of 8 compounds in 16 of 29 tumor types considered (Table 6).

**Table 5:** Summary of clinical benefit rate (SD/PR/CR, 16 weeks)

	Buparlisib (BKM120)	Dovitinib (TKI258)	Binimetinib (MEK162)	Encorafenib (LGX818)	Sonidegib (LDE225)	BGJ398	Ceritinib (LDK378)	Ribociclib (LEE011)	Total
Dosed patients, n	146	80	110	12	10	82^a^	47	106	593^a^
Clinical benefit, n (%)	22 (15.1)	11 (13.8)	25 (22.7)	3 (25.0)	0	12 (14.6)	9 (19.1)	19 (17.9)	101 (17.0)

Abbreviations: CR, complete response; PR, partial response; SD, stable disease.

^a^ Excluded treatment-induced osteomalacia–only patients.

**Table 6:** Summary of observed responses (CR + PR)^a^

Agent	Tumor type	Mutation	Prior lines of therapy, n	Best response	Treatment duration, weeks	Response confirmed (Yes/No)^b^	Age, years
BGJ398	Ovarian	FGF23 and FGF6 ligand amplifications	8	PR^c^	9.9	No	63
BGJ398	HNSCC	FGF19, FGF4, FGF23, FGF3, and FGF6 ligand amplifications	3	PR	63	Yes	49
BGJ398	HNSCC	*FGFR3* mutation; FGF3, FGF4, and FGF19 ligand amplifications	Unknown	PR	16	Yes	59
BGJ398	Tumor-induced osteomalacia^d^	*FGFR1* translocation	0^e^	PR	77.9	Yes	62
BGJ398	HNSCC	FGF3, FGF4, and FGF19 ligand amplifications	4	PR	21.6	No	71
BGJ398	CNS	*FGFR3* amplification; *FGFR3* fusion	1	PR	71.9	Yes	45
BGJ398	Ovarian	*FGFR1* amplification	4	PR^c^	6.3	No	73
BGJ398	NSCLC (squamous)	*FGFR1* mutation	4	PR	30.4	Yes	72
BGJ398	Bladder	*FGFR3* mutation	3	PR^c^	12.9	No	80
BGJ398	HNSCC	FGF3, FGF4, and FGF19 ligand amplifications	1	PR^c^	15	No	56
BGJ398	NSCLC (squamous)	*FGFR2* mutation	2	PR	96.6^f^	Yes	74
LDK378	Lung, non-small cell adenocarcinoma	*ROS1* rearrangement	4	PR	29.3	Yes	81
LDK378	Lymphoma	*ALK* mutation	3	PR	102.7^f^	Yes	22
LDK378	Lung, non-small cell adenocarcinoma	*ROS1* rearrangement	3	PR	23.9	Yes	58
BKM120	Cervical	PTEN loss (by IHC)	4	PR	24	No	57
BKM120	Vaginal	*PIK3CA*	1	CR	7.6	Yes	68
BKM120	HNSCC	*PIK3CA* and *PIK3CA* amplification	1	PR	32.3	Yes	65
TKI258	Ovarian	*FGFR2*	5	PR	32.7	No	74
TKI258	GIST	*cKit*	3	PR	33.1	Yes	49
MEK162	AML	*NRAS*	1	CR	20	NA	68
MEK162	Ovarian	*KRAS* (*KRAS* amplification and *KRAS G12D* mutation)	5	PR	44.3	Yes	78
MEK162	Ovarian	*KRAS*	3	PR	15.9	No	45
MEK162	Thyroid	*NRAS*	1	PR	36	Yes	75
MEK162	Uterine	*KRAS* (*G12V*)	7	PR	21.6	Yes	59
MEK162	Myeloma	*KRAS*	2	Very good PR	16.6	NA	59
LEE011	Bladder	*CCND1* amplification	2	PR	43	Yes	53
LEE011	Ovarian	*CDK6* mutation	2	PR	38.1	No	66
LEE011	Sarcoma	*CDK4* amplification	1	PR	53.7	Yes	25
LEE011	Unknown primary	*CDK6* amplification	1	PR	153.1^f^	Yes	59

Abbreviations: ALK, anaplastic lymphoma kinase; AML, acute myeloid leukemia; CCND, cyclin D; CDK, cyclic-dependent kinase; CNS, central nervous system; CR, complete response; FGF, fibroblast growth factor; FGFR, fibroblast growth factor receptor; GIST, gastrointestinal stromal tumor; HNSCC, head and neck squamous cell cancer; IHC, immunohistochemistry; KRAS, Kirsten rat sarcoma viral oncogene homolog; NA, not applicable; NRAS, neuroblastoma RAS viral oncogene homolog; NSCLC, non-small cell lung cancer; PIK3CA, phosphatidylinositol 4,5-bisphosphate 3-kinase catalytic subunit α; PR, partial response; PTEN, phosphate and tensin homolog.

^a^All patients experiencing a CR or PR at any time are included. Note that not all responses were confirmed responses by Response Evaluation Criteria In Solid Tumors or other related criteria.

^b^Unconfirmed response; patients may not have had a confirmatory scan due to protocol deviation or withdrawal from the trial before the confirmatory scan.

^c^Response not > 16 weeks.

^d^As defined by protocol.

^e^Patient had not been treated with prior chemotherapy/medication, but had 1 prior radiotherapy and surgery.

^f^Patient was still on study at time of data cutoff.

As an example, one responder, an 87-year-old woman with poorly differentiated colon adenocarcinoma and peritoneal carcinomatosis with anaplastic lymphoma kinase (*ALK*)–striatin calmodulin-binding protein (*STRN*) fusion (as well as genetic alterations in *KRAS,* serine/threonine kinase 11 [*STK11*], and *TP53*), was treated with ceritinib [7]. This patient was previously treated with 16 cycles of FOLFOX (leucovorin, 5-fluorouracil [5-FU], oxaliplatin), 8 cycles of FOLFIRI (leucovorin, 5-FU, irinotecan), and 6 cycles of 5-FU, and had progressive disease. At the data cutoff, the patient remained on ceritinib after > 31 weeks of treatment. Peritoneal masses were stable in size, and a mass protruding through the skin from the periumbilical node had regressed. Another patient experienced an unexpected complete response to the mitogen-activated protein kinase/extracellular signal–regulated kinase kinase (*MEK*) inhibitor binimetinib. This 70-year-old man had acute myeloid leukemia with *NRAS* mutations (G12D, Q61R, E62K), and had received 1 prior line of therapy.

## DISCUSSION

The Signature Program is an industry-sponsored series of compound-specific protocols designed to operationalize an approach for rapid signal finding that could potentially improve patient outcomes and increase efficiencies for further development of the compounds involved. The program spanned the spectrum of research-qualified practices—from research-oriented community practices to academic investigators—with the program design allowing for a rapid study start-up at each treatment site. For instance, the mean start-up time was 5.9 weeks (median, 3.6 weeks) compared with 10.4 months for traditional phase 2 to 4 oncology trials [8]. Although not a direct comparison, this short start-up time allowed patients to be treated promptly and the efficacy of the drug to be assessed quickly.

Patients were matched with a targeted agent that was predicted to result in clinical benefit regardless of tumor type. Responses were seen in some patients despite these patients having exhausted standard therapy options (≥ 3 in some patients) or having no other available treatment options. Overall, the program provided treatment options to a patient population with advanced disease and few to no available treatment options. Although the response rate was not high, our findings are consistent with those of the recently published MOSCATO-01 trial and preliminary data from the ongoing ProfilER study, which used high-throughput genomics to select therapies for difficult-to-treat advanced cancers [9, 10]. As was observed in our study, alterations in *RAS*, *PIK3CA*, *CDKN2A,* and *PTEN* were the most commonly observed across a range of solid tumor types included in the ProfilER study. Similarly, only a minority of patients with actionable genomic alterations received matched targeted therapies in both studies (MOSCATO-01: n = 199 of N = 411; ProfilER: n = 101 of N = 644), and modest clinical benefits were observed, with objective responses in 11% and 15% of evaluable patients, respectively. It is likely that expanding access to targeted anticancer agents could increase the proportion of patients who could benefit from basket protocols.

The Signature Program offered several advantages over a traditional study design. The program provided an opportunity to share emerging efficacy and safety data more quickly while minimizing the logistic challenges typical of standard clinical trials. Moreover, the patient-centric design of the program provided faster access to treatment, allowed patients to remain close to home at their current point of care, and permitted community research physicians to continue providing care rather than referring patients to a distant clinical trial center. Also, patients were preidentified based on local molecular profiling rather than undergoing central molecular prescreening. Furthermore, the adaptive statistical design required fewer enrolled patients to assess a signal, as data were analyzed for futility or efficacy more frequently rather than at the end of the study.

Another important element of this approach is that it eliminated nonenrolling sites—one of the operational inefficiencies common to site-centered cancer clinical trials. Currently, it is estimated that 20% to 30% of study sites across clinical trials enroll no patients [11]. Similarly, among Novartis-sponsored studies in the United States over the past 2 years, nearly one-third of opened sites failed to enroll any patients (data on file). With the cost of opening a study site estimated to be ≈ $50,000, regardless of whether any patients are enrolled [12], nonenrolling sites are a substantial burden on trial budgets and timelines. By limiting study participation to sites that had a potentially eligible patient ready to enroll, the Signature Program offered a more efficient and patient-centric approach that minimized this burden.

An operational challenge was to ensure a rapid and robust site-qualification process that would protect the quality of the research and data while enabling rapid site start-up. However, the program design overcame this challenge and led to a diversity of sites including academic institutions, community sites, and research networks across all states, which resulted in accelerated accrual timelines. For example, 13 cohorts reached the target accrual to allow for futility analysis in an average of 9.1 months. Four of these cohorts closed due to futility (Table 4), whereas the remaining cohorts stayed open until those studies were no longer open for enrollment.

Given that some gene alterations occur across different tumor types, the basket trial approach is driven by the interest in rapidly focusing an early drug candidate to the area(s) of greatest benefit, regardless of the overlying tumor. This approach is being used in several ongoing trials sponsored in both academia and industry. Although different in design to the Signature Program, implementation of a tissue-agnostic, molecularly driven approach led to the recent approval of pembrolizumab for the treatment of patients with any solid tumor exhibiting a specific biomarker (microsatellite instability-high or mismatch repair deficient). This is the first time that approval of a cancer treatment was based on the presence of a biomarker instead of the tumor's primary location [13]. Another example is the recently published study exploring vemurafenib in nonmelanoma cancers that specifically contain *BRAF* V600 mutations [14]. Within the 9 arms in the study, most of the 122 patients had NSCLC, colorectal cancer, or Erdheim-Chester disease and Langerhans cell histiocytosis. In both NSCLC and Erdheim-Chester disease, the authors identified potential signals for further investigation with objective complete and partial response rates of 42% and 43%, respectively. Even more recently, early results from the MyPathway study, evaluating the efficacy of 4 targeted treatments in 35 different tumor types with activating molecular alterations in the human epidermal growth factor receptor 2 (HER2), BRAF, epidermal growth factor receptor (EGFR), or Hedgehog pathways, were reported [15]. Of the 230 treated patients evaluated for a response (or discontinuing treatment prior to evaluation), 23% had an objective response (complete or partial response). All 4 treatments produced meaningful responses, with particularly notable objective response rates in patients with human EGFR-2–amplified/overexpressing colorectal tumors (38%) and BRAF V600-mutated NSCLC (43%).

Collaborative genomically driven basket studies include the ongoing ASCO Targeted Agent and Profiling Utilization Registry (TAPUR) study and the NCI-MATCH trial [16, 17]. The TAPUR study, which opened in March 2016, involves several pharmaceutical companies and consequently includes more arms than the Signature Program. Based on initial data, expansion of 4 cohorts and closure of a further cohort was recommended. The NCI-MATCH trial, which will enroll up to 6000 patients, opened in August 2015 with 10 arms and showed remarkably fast accrual of patients for molecular profiling, thereby demonstrating the interest in precision oncology among patients and physicians. However, the study suspended recruitment due to challenges in matching sufficient numbers of profiled patients to targeted therapies. The study reopened with 24 treatment arms.

The Signature Program contrasts with the NCI-MATCH study in several ways. First, NCI-MATCH requires a fresh biopsy with molecular characterization, whereas Signature allowed enrollment based on local testing of archival or fresh tissue, and only allowed patients to enroll after it was confirmed their tumor alteration was relevant to a specific protocol. Although obtaining a proximal tumor sample is theoretically appealing, because it minimizes the impact of genomic evolution, it has the clear disadvantage that most patients who wait for tissue characterization may not “match” to a treatment arm. Further, the statistical designs used in the 2 studies differed, with the Signature Program using a modified Bayesian adaptive design with a hierarchical model, allowing for dynamic borrowing of information, across tumor types within each of its basket trials. Critically, while the NCI-MATCH study does not use a Bayesian design, the Signature Program uses an improvement of the Bayesian hierarchical model. The method clusters tumor types such that those within the same cluster contribute more strongly to each other than do those outside the cluster. Clusters may be defined in advance, but an adaptive design also allows for clusters to be established based on similar outcomes, thereby increasing the odds of clinical benefit and enabling more signal finding [6, 18].

The Bayesian approach has been increasingly employed in all phases of drug development [18–23], and the specific advantages of the Bayesian design used in the Signature trials have been previously described in detail [6, 24]. In brief, Berry [6] compared the performance of the adaptive Bayesian hierarchical approach used in the Signature Program with that of the standard inferential and design approach used in the NCI-MATCH and vemurafenib trials, and determined that adaptive and hierarchical borrowing contribute to the accuracy and efficiency (smaller sample size) of indication finding in the Signature Program. Thus, using basket trials in the manner described has the potential for added efficiency in drug development. While the differential responses in patients may be due to tumor heterogeneity, rapid tumor adaptation to target inhibition, or other tissue-specific mechanisms, the strength of the signal (or lack thereof) in small tissue-specific cohorts interrogated through adaptive designs provides important clues to guide investigators toward clinical settings in which treatment relevance is most easily determined. Given the substantial cost of downstream development, which can be as high as $2.8 billion [25], the ability to rapidly focus resources on the most promising clinical setting(s) can be expected to not only speed development, but also reduce overall costs. Whether this promise can be realized will depend on effective implementation of this type of signal-generation approach.

Several limitations of our approach should be considered. The most important limitation is that single-agent treatment with targeted agents has often produced either limited efficacy or limited duration of response. This limitation may be overcome by using combination therapy with a second compound either directed at another part of the pathway or with a different mechanism of action, such as an agent that is synergistic, additive, or concurrent chemotherapy. Similar trial designs could be used to interrogate the value of various combination approaches across multiple tumor types. A second potential limitation is the rarity of some mutations across the spectrum of most tumors. Mutation rates within tumor types remain an important covariate for patient accrual, with mutations of interest often occurring in a small fraction of any specific tumor type. Although the Signature platform expanded the ability to capture patients with mutations of interest from diverse sites (eg, academic institutions and community sites) and from across the country, a very low mutation rate in a very limited number of tumor types remained a limiting factor, as seen in the sonidegib protocol, in which only 10 patients were accrued over 11 months. A third concern is that weaker, but potentially useful, signals could be missed in the context of small cohorts and heterogeneous populations. However, having a threshold for signal detection results in focusing limited resources on areas that are likely to be of greatest overall clinical benefit. Lastly, given the noncomparative nature of these single-arm protocols, it is not possible to describe the true clinical efficacy, or lack thereof, and thus interpretation of clinical responses should be made with caution. Tissue-specific studies with appropriate comparator arms would be required if early signals of clinical benefit are detected.

The Signature Program was a successful approach. It led to rapid signal finding, reduced patient exposure to toxicity, substantially shortened trial start-up times compared with conventional approaches, and cost-savings as a result of not opening multiple unnecessary trials and avoiding the cost-burden of nonenrolling and nonaccruing sites. The success of the Signature Program has led to conventional pivotal and exploratory studies in single indications incorporating several of the program features, including rapid start-up of nonpreselected sites. Each site was created when a patient with an actionable alteration was recommended for participation in the study. This concept highlights one of the most important advances in clinical trial methodology, that is, the broadening of the application of randomization outside typical venues for clinical trials [26]. Our evolving knowledge of cancer biology underscores the need for a paradigm shift in the traditional clinical trial approach [6]. The Signature Program highlights one way that the clinical trial is changing and serves as a sign of the eventual merger between clinical research and clinical practice. Subsequent Signature studies using combination therapies, predicted to be successful based on additional preclinical data, will address some of the program limitations and help determine the role of such an approach in drug development.

## MATERIALS AND METHODS

### Study design

Each protocol evaluated the efficacy and safety of 1 agent (buparlisib, dovitinib, binimetinib, encorafenib, sonidegib, BGJ398, ceritinib, or ribociclib) in patients with any solid tumor or hematologic malignancy that had an actionable genetic alteration, as assessed by a local Clinical Laboratory Improvement Amendments–certified laboratory, and disease that had progressed on or after standard treatment. All protocols had a similar study design (Figure 1).

Once an actionable genetic alteration was identified, the research physician contacted Novartis and a start-up package for the relevant protocol (including a fixed contract, central institutional review board [IRB]-approved protocol, standard budget, and set of standard informed consent forms) was sent. Institutions residing in the United States and having conducted industry research in the last year were considered to be research-qualified sites and were eligible for participation. The selection of an actionable alteration was left to the discretion of the investigator; however, Novartis was available for consultation if needed. Patients with > 1 actionable mutation were enrolled in a trial for the relevant mutation(s) at the investigator's discretion. A cohort for a particular tumor type was formed after ≥ 4 patients with that tumor type were enrolled.

A tumor tissue sample (archival or fresh) was submitted to Novartis upon patient enrollment for post hoc central profiling in a panel of > 288 cancer-related genes, which was followed by a confirmatory test against a larger panel of genes.

Patients initiated treatment once eligibility criteria were confirmed by Novartis. Enrollment requirements were sponsor approval, a prescreening phone call, and completion of proper regulatory forms. Local IRBs were not used, and certain local IRBs required a waiver-of-jurisdiction form. Following an expedited site initiation visit, the protocol was opened for the preidentified patient and for future accrual at the site. Patients received treatment until disease progression, unacceptable toxicity, death, or discontinuation from study treatment.

### Eligibility criteria

Eligible patients must have had received ≥ 1 prior treatment and have had no remaining standard treatment options. Tumor types for which the agent has shown no benefit and those for which key studies are planned or ongoing were excluded.

### Endpoints and assessments

The primary endpoint of each study was the clinical benefit rate (defined as complete response, partial response, or stable disease) at 16 weeks per investigator assessment, Response Evaluation Criteria In Solid Tumors v1.1, or appropriate hematologic criteria. Secondary endpoints included response rate, duration of response, progression-free survival, and overall survival. Exploratory endpoints included correlations between local and central molecular profiling data to evaluate the mutational pathway status of complementary DNA and the relationship to the response to treatment. Preliminary safety and tolerability results were also collected.

### Statistical analysis

To produce a higher statistical power (and lower risk of type I error) with fewer patients, we evaluated outcomes using a patient-sparing, Bayesian hierarchical model with clustering and dynamic borrowing [6, 27]. Cohorts with similar historical response rates were clustered using a Dirichlet process mixture model. Historical response rates were determined by examining published overall response rate data for patients who had received several lines of standard-of-care therapy. Hierarchical models were then placed over the cohorts within each cluster to determine the appropriate extent of borrowing between cohorts. Because the model does not allow borrowing across clusters, borrowing across dissimilar subgroups was minimized by assigning cohorts with dissimilar responses to different clusters.

Interim analyses were conducted to evaluate early futility and success in cohorts with a minimum of 10 patients at 16 weeks. A lack of clinical improvement in ≥ 10 patients within a cohort established futility. Clinical futility was defined as < 10% probability that the response rate in a group exceeded the historical rate. Clinical success observed in a cohort of 15 to 30 patients, depending on the strength of the response signal within the cohort and in the other cohorts for that compound, confirmed a positive signal. If there was ≥ 95% probability that the response rate in a group exceeded the historical rate, observed in ≥ 15 patients within a cohort, enrollment was stopped early for success.

### Ethical oversight

This clinical study was designed, implemented, and reported in accordance with the International Conference on Harmonisation–Harmonised Tripartite Guidelines for Good Clinical Practice, with applicable local regulations, and the Declaration of Helsinki. The study protocol and proposed informed consent form were reviewed and approved by a central IRB (Quorum) before study start. All patients provided informed consent.

## Abbreviations

5-FU: 5-fluorouracil
Adeno: adenocarcinoma
ALK: anaplastic lymphoma kinase
ALL: acute lymphocytic leukemia
AML: acute myeloid leukemia
CCND: cyclin D
CDK: cyclin-dependent kinase
CDKN2A: cyclin-dependent kinase inhibitor 2A
CLIA: Clinical Laboratory Improvement Amendments
CML: chronic myeloid leukemia
CNS: central nervous system
CR: complete response
CSF-1R: colony-stimulating factor 1 receptor
ECOG: Eastern Cooperative Oncology Group
ER: estrogen receptor
FGF: fibroblast growth factor
FGFR: fibroblast growth factor receptor
FLT3: fms-related tyrosine kinase 3
FOLFOX: leucovorin, 5-fluorouracil, oxaliplatin
GE: gastroesophageal
GIST: gastrointestinal stromal tumor
HNSCC: head and neck squamous cell carcinoma
HRAS: Harvey rat sarcoma viral oncogene homolog
IHC: immunohistochemistry
IRB: institutional review board
KDR: kinase insert domain receptor
KRAS: Kirsten rat sarcoma viral oncogene homolog
MEK: mitogen-activated protein kinase/extracellular signal–regulated kinase kinase
NF1: neurofibromatosis type 1
NRAS: neuroblastoma RAS viral oncogene homolog
NSCLC: non-small cell lung cancer
NTRK1: neurotrophic tyrosine kinase receptor type 1
PDGFR: platelet-derived growth factor receptor
PI3K: phosphatidylinositol 3-kinase
PIK3CA: phosphatidylinositol 4,5-bisphosphate 3-kinase catalytic subunit α
PIK3R1: phosphatidylinositol 3-kinase regulatory subunit polypeptide 1
PR: partial response
PS: performance status
PTCH1: patched 1
PTEN: phosphatase and tensin homolog
RTK: receptor tyrosine kinase
SIV: site initiation visit
SMO: smoothened
TrkA: tropomyosin receptor kinase A
VEGFR: vascular endothelial growth factor receptor
